# Antimicrobial Use and Resistance: Knowledge, Attitudes, and Practices of Broiler Farmers in Mzimba, Malawi

**DOI:** 10.3390/antibiotics15030239

**Published:** 2026-02-25

**Authors:** Abel Compbel Chipembo, Goliath Eneya Zulu, Precious Innocent Mastala, Sam Mvula, Thomas S. G. Malinki, Wilson Friday, Martin Kalumbi, Alberto Pondja, Janelisa Musaya, Belisário Moiane, Niura Madalena Bila

**Affiliations:** 1 Department of Animal Production and Food Safety, Faculty of Veterinary Sciences, Eduardo Mondlane University, Bairro Luis Cabral, Avenida de Moçambique, Km 1.5, Maputo P.O. Box 257, Mozambique; goliathzulu@uem.ac.mz (G.E.Z.); apondja@uem.mz (A.P.); belisario.moiane@uem.mz (B.M.); 2 Centre of Excellence in Agri-Food Systems and Nutrition (CE-AFSN), Eduardo Mondlane University, Praça 25 de Junho Posta 257 Edificio da Reitoria 5° Andar, Maputo P.O. Box 257, Mozambique; thomasmalinki@uem.ac.mz; 3 Department of Epidemiology and Biostatistics, Mel and Enid Zuckerman College of Public Health, The University of Arizona, 1295 N. Martin Ave., Tucson, AZ 85724, USA; pmastala@arizona.edu; 4 Mzuzu Regional Veterinary Laboratory, Mzuzu P.O. Box 8, Malawi; sam.mvula@agriculture.gov.mw; 5 Department of Chemical Engineering, Faculty of Engineering, Eduardo Mondlane University, Avenida de Moçambique, Km 1.5, Maputo P.O. Box 257, Mozambique; 6 Department of Agribusiness and Natural Resources Economics, College of Agricultural and Environmental Sciences, Makerere University, Kampala P.O. Box 7062, Uganda; friday.wilson@student.mmu.ac.ug; 7 Department of Biomedical Sciences, Malamulo College of Health Science, Makwasa P.O. Box 55, Malawi; kalumbim@mchs.adventist.org; 8 Malawi Liverpool Wellcome Trust, Chichiri, Blantyre P.O. Box 30096, Malawi; jmusaya@kuhes.ac.mw

**Keywords:** antimicrobial resistance, antimicrobial use, awareness, chickens, antimicrobials, Malawi

## Abstract

**Background**: The use of antimicrobials in chicken is well-known worldwide. However, the motivating factors towards the use of antimicrobials by poultry farmers are not well-known. Furthermore, awareness of antimicrobial resistance and how such factors can lead to AMR in bacterial poultry pathogens, particularly those isolated from chicken meat in Mzimba district, is not well-documented. **Objective**: To evaluate the knowledge, attitudes, and practices about the use of antimicrobials among chicken farmers in the Mzimba district, Malawi. **Materials and Methods**: A cross-sectional study was conducted involving 89 chicken farmers in Mzimba to assess their knowledge, attitudes, and practices (KAP) related to AMU and AMR. Data analysis was performed using STATA version 15, applying linear regression and Pearson correlation analysis for KAP scores. **Results**: Among the chicken farmers, 46.1%, 43.8%, and 42.7% demonstrated good knowledge, attitudes, and practices, respectively. Training on AMU and AMR was significantly associated with knowledge (*p* = 0.002) and practices (*p* = 0.02). There were weak relationships among knowledge, attitudes, and practices scores, with *p*-values of 0.2, 0.07, and −0.05. There were gaps in coordination between veterinarians and farmers, and all farmers (100%) obtained antimicrobials from veterinary shops without consulting veterinarians. Farmers were not aware of policies related to AMR and AMU. **Conclusions**: Chicken farmers exhibited low KAP levels on AMU and AMR. This situation constitutes an emergency of AMR and therefore underscores the need for policy development targeted towards enforcing regulations and improving KAP through trainings programs on AMU, AMR, and the importance of consulting veterinarians in chicken production.

## 1. Introduction

The poultry sector is expanding rapidly worldwide and is an increasingly important source of affordable animal protein [1,2]. In sub-Saharan Africa, poultry production and consumption are rising, driven by population growth, urbanization, and changing diets [3]. Malawi has also experienced growth in poultry production and trade, with chicken meat and eggs contributing to household nutrition and income generation [2,4].

As poultry systems intensify to meet growing demand, farmers and producers often prioritize faster growth, improved feed conversion, and disease prevention. In many settings, this has been accompanied by increased antimicrobial use to manage and prevent bacterial infections [5,6,7,8]. However, when antimicrobials are used without appropriate prescription, oversight, or adherence to recommended dosages and withdrawal periods, selective pressure promotes the emergence and spread of antimicrobial resistance (AMR). AMR is a One Health challenge because resistant bacteria and resistance genes can move between animals, people, food products, and the environment, undermining treatment options and increasing the cost and complexity of care [9].

Several studies have reported that in Low- and Middle-Income Countries (LMIC) like Malawi and Zimbabwe, most farmers have limited knowledge, attitude and practices (KAP) on antimicrobial use and resistance [8,10,11,12]. On top of that, evidence suggests that antimicrobial use in livestock is frequently shaped by limited access to trained veterinary services, weak regulation and enforcement, and reliance on advice from veterinary drug shop personnel who may be inadequately trained [13,14,15,16]. These factors can contribute to inappropriate antimicrobial selection, under-dosing, prolonged use, and routine prophylaxis. At the same time, poultry husbandry and biosecurity practices may be suboptimal, increasing infection pressure and further incentivizing antimicrobial use [8,14].

The majority of chicken farmers in Malawi lack proper training in poultry husbandry, including biosecurity and the responsible use of antimicrobials [8]. Currently, there are no recommendations regarding the prudent use of antibiotics in poultry and food animals. The limited awareness and knowledge of antimicrobial use among the majority of poultry farmers can lead to inappropriate use of antimicrobials [16].

Recent studies in Malawi have reported antimicrobial-resistant bacteria in food animals and food products, raising concerns for food safety and public health [17,18]. The consumption or handling of poultry contaminated with resistant bacteria such as *Escherichia coli* and *Salmonella* may lead to infections that are harder to treat. Inadequate observance of withdrawal periods may also result in antimicrobial residues in edible tissues, posing additional risks to consumers [14]. Despite these concerns, the local drivers of antimicrobial use and the burden of AMR in Malawi remain insufficiently characterized, particularly among small-scale poultry farmers in rural districts.

The Mzimba district is found in the northern region of Malawi. The district is predominantly rural, characterized by small-scale poultry production and limited veterinary service provision. In such contexts, farmers’ knowledge, attitudes, and practices (KAP) regarding antimicrobial use and resistance are likely to influence antimicrobial-use behaviors and the effectiveness of any stewardship interventions. In addition, anecdotal reports indicate that some farmers may use herbal remedies to treat poultry illnesses, but the patterns of use and perceived effectiveness are not well-described.

Therefore, this study aimed to assess the knowledge, attitudes, and practices of poultry farmers in the Mzimba district regarding antimicrobial use and antimicrobial resistance. Understanding these factors is essential for designing targeted training, strengthening antimicrobial stewardship, informing policy and guidelines for prudent antimicrobial use in food animals, and ultimately reducing the risk of AMR at the human–animal interface.

## 2. Results

### 2.1. KAP Survey Results

#### 2.1.1. Demographic Information

The study conducted a total of 89 interviews with farmers (Table 1). The interview results showed that the majority of smallholders were males (51%) with ages ranging from 31 to 40 years (30.3%). In addition, 70% (n = 62) of the chicken farmers had completed high school, at least 74% had never received formal training specifically in AMR or antibiotic use, and 32% (n = 32) of the breeders had less than 5 years of experience raising broilers.

#### 2.1.2. AMU Situation Analysis Among Chicken Farmers

**Figure 1:**
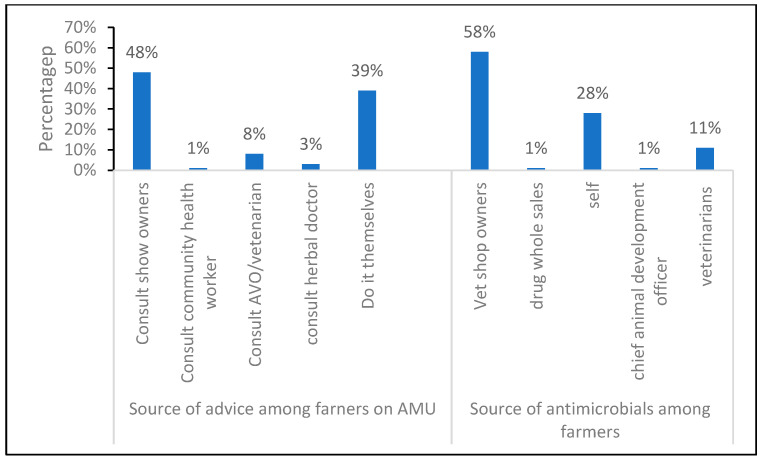
Information on the source of antimicrobials and advice on AMU among farmers.

Most farmers relied on veterinary shop owners for advice on antimicrobial use, for a total of 58% of respondents. In contrast, 28% were relying on their own knowledge; only 11% were seeking advice from veterinarians. When it comes to sourcing antimicrobials, 89% were purchasing from agrovets instead of veterinarians and the chief animal development officer (Figure 1).

**Figure 2:**
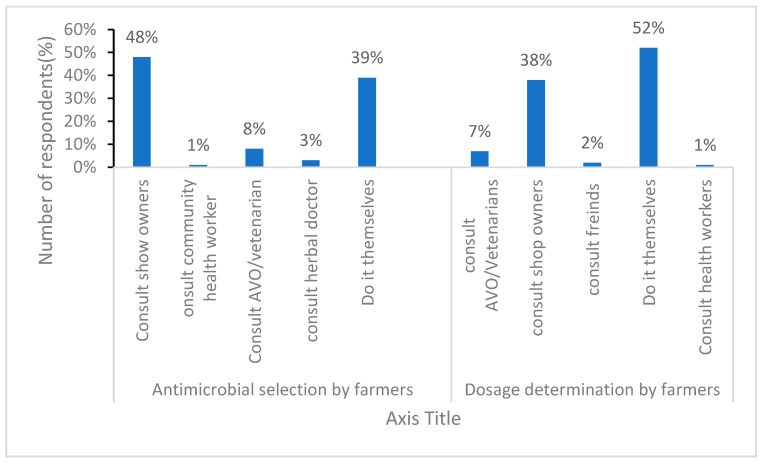
Information on selection and usage of antimicrobials among farmers.

When it comes to selecting antimicrobials, the majority of the farmers (49%) indicated that they consulted shop owners, whereas a small proportion sought guidance from veterinarians and community health workers. For determining dosages, 52% (n = 46) relied on their own judgment, 38% (n = 34) consulted shop owners, and 7% (n = 6) sought advice from Animal Vaccinators (AVOs) (Figure 2).

#### 2.1.3. Information on Antimicrobial Prescription and Use

Seventy-six percent of farmers were aware that veterinarians have the authority to write prescriptions. However, 90% of respondents reported that veterinarians do not actually write prescriptions. Additionally, 97% of respondents indicated that veterinary shops do not require prescriptions. In terms of accessing antibiotics, 92% (n = 82) purchased them from shops without prescriptions, while 6% (n = 5) purchased them from shops with prescriptions, 2% (n = 2) sourced antibiotics from other shops, and 5% from shops that required prescriptions (Table 2).

To evaluate how AMU is monitored in chicken production, farmers were asked to identify the responsible individuals overseeing AMU on their farms. According to the responses, 93.3% of the farmers indicated that the Department of Animal Health and Livestock Development (DAHLD) was responsible for monitoring AMU. Additionally, 31% reported that agrovets shops play a role in checking AMU on farms, while 20% indicated that the Malawi Pharmacy and Medicines Regulatory Authority is responsible. Furthermore, 11% cited the Malawi Bureau of Standards, and 6.7% reported that the Poison and Medicine Board is mandated to oversee this.

#### 2.1.4. Challenges Faced by Farmers to Continue the Use of Antimicrobials in Chicken Farming

**Figure 3:**
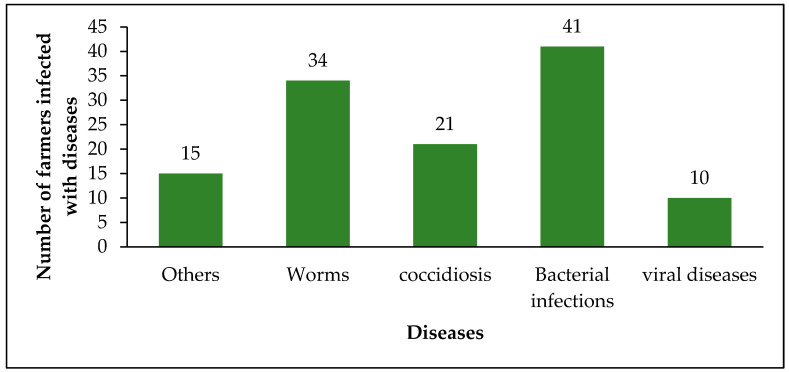
Diseases infecting chickens mentioned by farmers.

A high prevalence of chicken diseases was reported by 84.2% (n = 75) of respondents as the primary challenge forcing them to use antimicrobials. Additionally, 2.3% cited the limited availability of vaccines as a reason for antimicrobial use. Furthermore, 12.4% (n = 11) reported using antimicrobials to maintain productivity and profitability, while 1% reported using antibiotics due to difficulties in accessing veterinary professionals. Some of the diseases reported to be infecting chickens include viral infections, e.g., Gumboro disease, bacterial infections, worm infestations, viral diseases, and other unidentified illnesses (Figure 3).

In terms of antibiotics commonly used by farmers, most farmers reported tetracycline (63%), sulfonamides (22.5%), and quinolone (16.8%) (Table 3). In addition to conventional or modern antibiotics, 11.2% of farmers (n = 10) used natural herbal medicine to treat chicken infections. The following were some of the herbal antibiotics used by farmers that were reported: Guava extracts (*Psidium guajava*), Moringa, majick powder, Alouvela (*Aloe barbadensis miller*), avocado (*Persea americana*), eucalyptus, duhat (Java plum, *Syzygium cumini*), tamarind (*Tamarind indica*), and natural antibiotics (plant-derived compounds). Apart from herbal medicine, 6.7% farmers (n = 6) were using diaglow (a pesticide) as an antibiotic.

#### 2.1.5. Knowledge About National Policies Related to AMU and AMR

When the knowledge of farmers about national policies related to antimicrobial use in chicken farming was assessed, 93.2% (n = 83) of the farmers were not aware of national policies, while 6.8% (n = 6) farmers were aware of national policies. The farmers who were aware of national policies had attended secondary schools and tertiary institutions. The farmers who reported that they knew national policies mentioned that they do not follow such policies. While the farmers who reported that they do not know national policies stated that most of them do not know such regulations, others reported that people do not follow laws, and others said the government is not implementing such laws related to AMU. Farmers who reported that they know national policies related to antimicrobial use and AMR provided the following recommendations: providing increased access to antimicrobials and increasing farmers′ training and awareness.

#### 2.1.6. Knowledge of Chicken Farmers About AMU and AMR

Mean scores across all 11 questions were used as a cut-off point to assess the knowledge of AMU and AMR. The total mean score with its standard error was 9.4 ± 0.49. Using the total mean score as a cut-off point, only 46.1% of respondents demonstrated proficiency by providing correct answers above the mean of the questions, while the remaining 53.9% yielded responses falling below the mean.

Of the 89 farmers, 25% (n = 22 respondents) recognized AMR as a serious public health problem, while 75% (n = 67 respondents) did not. About 95% had heard about AMU and 98% had never heard anything about AMR. In regard to the knowledge of antimicrobial transfer via chicken products, 96% (n = 85 respondents) knew that antimicrobials can be transferred to the humans via chicken products, and 91% of them knew that inappropriate use of antimicrobials can lead to the development of AMR. Among 89 farmers, 97% reported that it is wrong to sell chicken meat before the withdrawal period, and 94% reported that antimicrobial residues could be serious to public health; however, 27% were aware that the antimicrobials use in feed formulation is inappropriate. Notably, 63% were aware of the institutions that control sales of the antimicrobials, and 70% were aware that the lack of control over the sales of antimicrobials can lead to AMR (Table 4).

The coefficient analysis revealed significant relationships between gender, education level, and training in AMR and AMU with farmers’ knowledge scores.

In contrast, age, marital status, and years of poultry farming experience were not significantly associated (Table 5).

#### 2.1.7. Attitudes of Chicken Farmers on AMU and AMR

The total mean score of respondents for a total of 14 questions was used as a cut-off point to assess the attitudes of respondents toward AMU and AMR. The total mean score with its standard error was 30.47 ± 49. Using this as a cut-off point, only 43.8% of respondents demonstrated proficiency by providing correct answers to over 30%% of the questions, while the remaining 56.2% yielded responses below the mean. On safety, 98% (n = 87) of the respondents strongly agree that antimicrobials are safe for humans and animals, and 91% (n = 81) strongly agree that antimicrobials are needed for any animal illness. About 93.26% (n = 83) of the respondents strongly agree that broad-spectrum antimicrobials are capable of curing any infection.

Out of 89 farmers, 82% (n = 73) strongly agreed that antimicrobials are needed to prevent serious illnesses, but only 35% (n = 34) strongly disagreed with adding antimicrobials to feed. A total of 94% of respondents strongly agreed that non-prescribed antimicrobial sales should be prohibited, 57% of the respondents strongly agreed that antimicrobials are needed during weather/season changes, and 63% strongly agreed that they are needed for fever/cold.

In regard to disease symptoms, 79% (n = 70) strongly agreed that stopping antimicrobials after symptoms disappear is safe to preserve them for the future, and 96% (n = 85) strongly agreed that missing doses contribute to AMR. In terms of the overdosing of antimicrobials, only 33% (n = 29) strongly agreed that overdosing could lead to AMR, with 39.3% (n = 35) strongly disagreeing, and 62% (n = 55) strongly agreed that vaccination can reduce antimicrobial use. Seventy-nine percent (n = 70) of the respondents strongly disagreed that it is not good to use expired drugs in treating chicken diseases, and 78% (n = 69) of the respondents strongly disagreed that it is safe to eat animals that die during the course of treatment (Table 6).

Coefficients analysis revealed that all the independent variables were not significantly correlated with the farmers’ attitudes on AMU and AMR (Table 7).

#### 2.1.8. Practices of Chicken Farmers About the Use of Antimicrobials

The total mean scores of a total of nine practice questions administered to poultry farmers regarding their practices on AMU were used as the cut-off point. The total mean score with its standard error was 4.4 ± 0.18. Using the total mean score as the cut-off point, 47.5% demonstrated proficiency by correctly answering the questions above the mean score of 4.4, while 59.3% exhibited responses that fell below this mean score.

Notably, 94% (n = 84) of the respondents reported that they treated chickens without conducting antimicrobial sensitivity tests, while 6% provided treatment after laboratory tests. It was also found that 51% reported not consulting anyone when chickens are sick. Apart from the consultation, 63% (n = 56) of the respondents selected antibiotics themselves, and 68% (n = 61) decided on the dosage themselves.

Despite the farmers administering drugs themselves, 91% (n = 81) reported that they allow their chickens to complete the full course of antimicrobial treatment when treated by veterinarians. Again, 84% (n = 75) reported that they check expiry dates. It was also discovered that 92% (n = 82) stop administering antibiotics when symptoms disappear. In terms of disposing of drugs, 92% (n = 82) mentioned that they burn or bury expired drugs, 64% (n = 57) reported that they throw them away, 6% (n = 5) put them in animal feed, 2% reported that they return them to the veterinary laboratory. Most farmers dispose of expired drugs appropriately, but a small proportion misuse them. It was also found that 80% of farmers increase the dose/frequency when clinical symptoms persist (Table 8).

Coefficients analysis between the independent variables (age, gender, marital status, years in practice, and education levels) was tested against the dependent variable (practices) using STATA. The findings revealed that only AMR training was significantly correlated with the practices (*p* = 0.024) (Table 9).

A correlation test between knowledge and attitudes, and between knowledge and practices was further assessed using Pearson’s correlation test. As per the criteria by [19], the correlations were ranked as 0–0.25 = weak correlation, 0.25–0.5 = fair correlation, above 0.5 = good correlation. Therefore, the correlation analysis between knowledge and attitude score indicated there was a weak positive relationship between knowledge and attitude indicated weak positive relation (r = 0.22). This means that the attitudes of the poultry farmers towards AMU and AMR were influenced by their knowledge. At the same time, knowledge about AMU and AMR influenced respondents’ attitudes, as shown in Table 10.

The correlation analysis between knowledge and practices score indicated that there was a weak relationship between knowledge and practices among participants in the study (0.07), suggesting that the practices of the poultry farmers towards AMU and AMR do not influence their knowledge. In contrast, the correlation analysis between practices and attitudes scores indicated there is a negative relationship between practices and attitudes among participants (−0.0539). This means that their knowledge influenced the attitudes of the poultry farmers towards AMU and AMR. At the same time, attitudes towards AMU and AMR influenced their practices (Table 10).

## 3. Discussion

### 3.1. AMU Situation Analysis

According to the authors, this is the first study to assess KAP among broiler farmers in relation to AMU and AMR in Malawi’s Mzimba district. The study revealed that the majority of the poultry farmers in Malawi obtain antimicrobials from agrovets and rely on veterinary shop owners for advice related to antimicrobial sources and selection of antimicrobials that are used in broiler chicken production. This is a similar scenario in other settings, such as in Bangladesh, Nigeria, and Fiji, where the majority of the poultry farmers obtain antimicrobials from feed sellers, and they also do not consult veterinarians for proper prescriptions [19,20,21,22]. This is largely due to economic reasons and dissatisfaction with the previous services offered by the government’s veterinary officers, which exacerbates AMR [19,20]. The results reveal high antimicrobial misuse, self-prescription, and reliance on agrovets shops, indicating that policies related to antimicrobial use are failures and that there is a lack of regulation related to accessibility to antimicrobials in chicken production.

The most commonly used antimicrobials from this study were comparable with the studies conducted in Malawi and other global settings [19,23,24,25]). For instance, tetracycline, sulphonamides, Oxytetracycline, and quinolones have been reported previously in Malawi, Bangladesh, Ethiopia, and Nigeria. However, quinolone use was not rampant in the present study compared to other settings. These differences suggest variation in geographical location, disease burden, management systems, economic related matters and distribution of the antibiotics. Interestingly, most of these antibiotics are mostly dispersed by veterinary shop owners as reported by the present study and others [19,23,24,25]. This implies that the distribution of these antibiotics is not satisfactory and the regulatory bodies must tighten the access to such antibiotics because these are similar antibiotics used in the treatment of human infections. The overuse of tetracyclines and other antibiotics in the poultry and other animal production increases the risk of AMR [26,27,28,29,30].

World Organization for Animal Health indicates that critical important antibiotics should be administered by a well-qualified person only [31]. Tetracyclines are classified as highly important antibiotics used in humans, while aminoglycoside, macrolides, polypeptides, and sulfonamides have been listed as important antibiotics [32]. It is inappropriate to utilize critically important antimicrobials for controlling the spread of clinically diagnosed infectious diseases within livestock or to treat livestock with clinically confirmed transmissible diseases.

WHO recommends that medically important antibiotics not be used in animals, which is not the case in Malawi, where most of the farmers use Bactrim and amoxicillin to treat infections in chickens [33]. These antibiotics are supposed to be reserved for the treatment of infections in humans. However, poverty drives the famers to use such antibiotics in chicken production with the aim of increasing the productivity of their enterprises. For instance, farmers reported that they are unable to buy recommended antibiotics to treat chickens because of financial problems. Others reported that they use Bactrim and amoxicillin because they get them for free from government hospitals. On top of that, other farmers were using antiretroviral (ARV) drugs for HIV/AIDS, which other farmers reported they could combine with Bactrim and give to chickens for growth promotion and as an antibiotic; this is a serious public health concern. Farmers using expired antibiotics is a serious public health concern. AMR is fueled by this type of antibiotic misuse and overuse. When antibiotics are used in broiler chicken treatment and do not follow the antibiotic withdrawal period, their residues are consumed by humans together with the meat. This promotes the development of bacterial antibiotic resistance (AMR) due to the inadequate concentration of antibiotics [23].

Although World Animal Protection bans routine antibiotic feeding to farm animals [24], in this study, most farmers reported using Egocine as a growth promoter as well as an antibiotic. Egocine contains oxy-tetracycline hydrochloride as the main antibiotic and water- and fat-soluble vitamins, which are used as routine growth promoters in chickens. This practice is not only unacceptable but is also against the action plan implemented by FAO, which prohibits the use of antibiotics as feed additives for livestock and poultry growth aimed at combating AMR [34]. Some nations have outlawed the use of antibiotics as growth promoters in livestock production, but Malawi is an exception.

This study shows that farmers’ judgment when choosing and determining the dosage of antibiotics can result in unintentional misapplication, including incorrect dosages, inappropriate treatment durations, and unnecessary use, which ultimately contributes to antibiotic resistance and poses risks to public health [35]. Farmers must follow internationally recognized guidelines, such as those set by the World Organization for Animal Health, to ensure the responsible use of antibiotics and prevent potential public health and food safety implications.

This investigation found that some farmers were using herbal remedies. The majority of farmers who acknowledged using these remedies said they do not use synthetic antibiotics. However, they are not sure of their dosages and withdrawal periods, because the pharmacokinetics and pharmacodynamics of the majority of these herbal medications are still unknown. Even so, results revealed that most farmers using herbal remedies on their chickens do not face bacterial infections. Although this is a promising solution, further research is needed to assess the efficacy and safety of the herbal medicines used by poultry farmers so that they can be a substitute for synthetic antibiotics. Some studies have reported that most bacteria are susceptible to natural remedies such as aloe vera and moringa [25,26], but more should be done to standardize the dosage and assess the residues of these herbal remedies in meat. In this study, it was also found that some farmers were using diagrow pesticide as an antibiotic; the use of pesticide can accumulate in chicken meat over time and can cause health problems in humans such as cancer and allergic reactions.

Understanding the use of antibiotics was one of the main problems portrayed in this study and elsewhere [27,28]. Farmers struggled to identify chicken diseases, although they were able to identify associated systems. This limits the ability to provide appropriate antibiotics for their chickens. But they indicated that they provide treatment based on the symptoms presented by chickens. Furthermore, symptom-based treatment alone is highly likely to result in misuse and overuse of antibiotics, which contributes to the development of AMR. The lack of knowledge regarding the specific indications and improper administration of antibiotics due to a lack of training in AMU increases the risk of antibiotic misuse and contributes to the development of MR in broiler chickens.

### 3.2. Knowledge, Attitudes, and Practices of Farmers Related to AMU and AMR

This study found a low knowledge of AMU and AMR, a negative attitude and poor practices towards AMU and AMR. The low level of knowledge could be associated with a lack of training in AMU and AMR. In this study, only a small proportion of the farmers were trained in AMU and AMR. This lack of appropriate training among poultry farmers is consistent with the findings reported in [12,22,23]. The majority of poultry producers lack sufficient expertise, as evidenced by the poor knowledge scores in this study. These farmers are unaware of the potential negative effects of improper use of antimicrobials, which can lead to the irrational use of these medications. Poultry producers who are ignorant about AMU and AMR run the risk of using antibiotics improperly, which exacerbates AMR [29]. Hence, there is a need for these poultry farmers to be sensitized regarding correct AMU and AMR.

The farmers’ low level of knowledge in AMR is concerning, especially when the majority indicated that they were not aware of AMR, which is a public health concern, and that antibiotics could be used to treat any disease. To these farmers, antibiotics can be used for any illness, including anti-parasitic, anti-protozoa, and viral infections. The low awareness of AMR among poultry farmers calls for improved educational interventions and the strengthening of antimicrobial stewardship programs [36].

In this study, education was shown to play a big role in knowledge on antimicrobial use and resistance among broiler farmers. A significant relationship was found between the knowledge level and education level. Most poultry farmers who attained tertiary education generally had high knowledge scores. This could be attributed to the fact that participants of the tertiary level and some from the secondary level may have learned about AMU and AMR or may have read about the same. Previous studies done in Sudan and Ethiopia reported similar findings that attributed the lack of knowledge on AMU and AMR to the level of education of the participants [30,37].

In this study, a serious gap was revealed in agricultural systems in Mzimba, Malawi, where a shortage of veterinarians leads to the administration of antibiotics by farmers without prescriptions and consultation. In some places, government veterinarians stopped visiting the farmers to assess the health status of their chickens. In areas where veterinarians are available, farmers’ lack of money to call veterinarians and loss of trust prevent them from accessing required services. Others reported that they have been doing chicken farming for years and claimed to have good experience in chicken farming that does not require the services of experts. Another fact that may be related to the failure to consult veterinarians is the ease of purchasing medications without a prescription, and easily accessible antibiotics in veterinary shops in Malawi [38,39]. In this study, most of farmers purchased over the counter antimicrobials without consulting veterinarians, which is also similar to studies conducted in Kenya, Tanzania, Serbia, Ghana and Nigeria, where farmers were buying over the counter antibiotics [38,39,40,41]. Normally, the procedure was supposed to be that the farmers should report disease cases to veterinarians, who would then diagnose the animals, purchase the drugs, and administer the recommended drugs to the animals [42]. Normally, veterinary shops are supposed to sell drugs on the basis of a prescription to registered veterinarians, rather than directly to farmers.

Farmers who lack expertise in chicken production and antibiotic use may not be aware of the appropriate use of antibiotics, including the correct dosage, duration, and withdrawal periods [43]. The lack of understanding among farmers, as evidenced by the use of antibiotics for various animal diseases without proper knowledge, raises immediate concerns about animal health and public health. Using antibiotics without understanding how to use them properly, and not considering other ways to treat illnesses can be risky and could lead to AMR [44]. The indiscriminate use of antibiotics without adequate knowledge can lead to ineffective treatments, prolonged illnesses, and economic losses. It is crucial to improve farmers’ understanding of antibiotic use and resistance to ensure responsible antibiotic usage and effective disease management practices in animals.

Interestingly, the majority of the farmers believed that the sale of non-prescription antibiotics should be outlawed because they frequently purchase expired medications, and some claim that antibiotics could not cure their chickens’ illnesses. Due to the high cost of the antibiotics, some farmers stop the dosage after the animal is cured and the antibiotics are reserved to be used in the future. The tendency to purchase non-potent and expired antibiotics could negatively affect the attitudes of farmers towards the use of drugs. The sale of non-potent and expired antibiotics is attributed to the lack of proper regulation of antibiotic use in Malawi. This increases the likelihood of using fake drugs or expired antimicrobials among farmers, which can lead to AMR, thus significantly affecting public health. Although farmers in the study reported obtaining antibiotics from registered shops, the absence of regulatory oversight in antibiotic acquisition contributes to unmonitored and possibly inappropriate use.

Poor practices regarding AMU and AMR reported in this study are another stumbling block to the efforts put in place by the Ministry of Agriculture in Malawi, WHO and other international organizations aimed at controlling AMR [45]. This is similar to the practices of farmers in Cambodia regarding AMU and AMR [46]. Treating chickens without antimicrobial sensitivity testing and consultations with veterinarians is one of the common practices that can influence AMR [8,11,13]. This is commonly due to the lack of diagnostic tools, shortage of veterinarians, and the high cost of sensitivity testing in Malawi, which hinders evidence-based treatment of bacterial infections in chickens. This might also be because most farmers are not trained in antibiotic usage and AMR.

There was a weak relationship between attitudes, practices, and knowledge. This study shows that despite people having knowledge on withdrawal periods and knowledge of antimicrobial resistance, they were not practicing such knowledge on the use of antimicrobials in chicken farming, which might contribute to antimicrobial resistance due to lack of training on AMU and AMR. This shows that a lack of training in AMU and AMR might be a contributing factor to low KAP. This study had a number of limitations. Firstly, only broiler farmers and one district were included in the study. The current study might not be representative of all of Malawi and all livestock animals, the short time span of the research may not reflect long-term dynamics, and also the sample was drawn from broiler farmers from February to May 2025, which limits the generalizability of the results to broader populations/contexts. Potential recall and social desirability bias may also affect the results, along with the lack of microbiological or residue analysis to complement self-reported practices, and the study’s cross-sectional design which limits causal inference. Nonetheless, the results of this study may offer a baseline comparison with the other districts of Malawi. Therefore, other studies must be conducted to assess KAP among other livestock farmers on AMU and AMR, the accessibility of antimicrobials, and the consultation of veterinarians on the use of antimicrobials.

## 4. Methodology

### 4.1. Study Area

**Figure 4:**
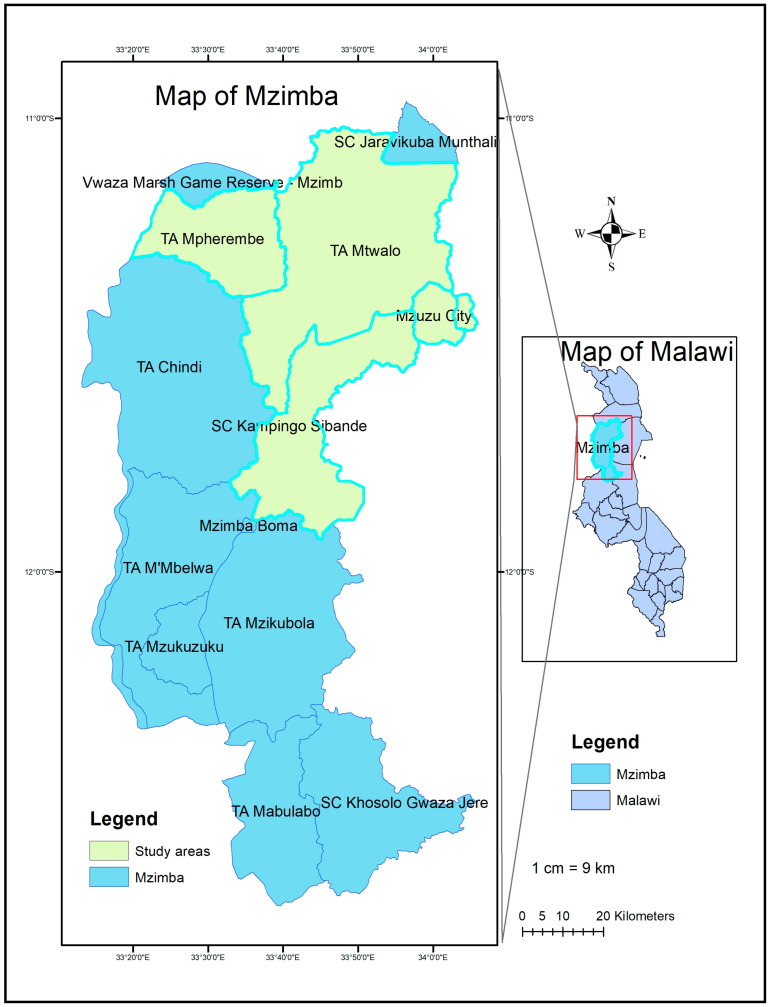
Study area: a map of Malawi showing the location of Mzimba district (highlighted in the green rectangle on the right, where yellow-color areas represent study areas). The map was drawn using ArcGIS Vision 15 Software, (Esris, Redlands, CA, USA).

The study was conducted in the northern region of Malawi, Mzimba district (Figure 4), targeting households of farmers keeping broiler chickens from February to May 2025. The area is located at Latitude: 11°54′0″ S and Longitude: 33°36′0″ E. It covers an area of 10,430 km^2^ and has a human population of approximately 610,944. Mzimba north includes areas such as Mzuzu, Ezondweni, Vwaza Wildlife Reserve, Ekwendeni, Mpherembe, and Mtwalo and Kampingo Sibande, which are notable for having a large population of people keeping chickens for both food and income (Figure 4).

### 4.2. Study Design, Study Participants, Sampling Method, and Sample Size

This study was conducted from February to May as a cross-sectional survey of broiler farmers in Mzimba. A comprehensive list of poultry farms was obtained from the district Animal Health Office (n = 197 farms). However, most farmers are seasonal and due to the high cost of feed, most farmers were not doing chicken farming during the time of data collection, and only 114 farmers were reported as active farmers during the study period. The targeted respondents were farm owners or managers. The sample size was calculated using OpenEpi software version 3, hypothesized at a 95% confidence level. The following formula was employed—*n* = [DEFF × Np (1 − p)]/[(d^2^/Z^2^_1−α/2_ × (N − 1) +p × (1 − p)]—where DEFF is Design effect: 1.0; N is population size: (the total number of household farmers keeping broilers in Mzimba North); d: confidence limits as % of 100 (absolute +/−%: 5%); p: hypothesized % frequency outcome in the population: 50% +/−5; and α = 0.05, Z = 1.96. The assumed prevalence of farmers using antibiotics was 50% of all farms. The total sample size calculated was 89 farms. Purposive sampling was used to target the calculated sample size that was active in chicken farming to evaluate antibiotic use and the farmer’s awareness on antimicrobial resistance. Potential participants were identified with the help of veterinary assistants, and a purposive and stratified sampling method was used to enroll chicken farmers based on the age group differences, education level, gender, marital status in the study, and farmers who have been trained on AMU and AMR.

### 4.3. Inclusion and Exclusion Criteria

#### 4.3.1. Inclusion Criteria

The study enrolled farmers residing in the aforementioned area, who were actively rearing chickens for meat during the time of the study and had provided consent. Those farmers with multiple animal types were also considered in this study, as long as they reared broiler chickens for commercial purposes.

#### 4.3.2. Exclusion Criteria

The study excluded all farmers who kept other animals, those farmers under 18 years of age, farmers who declined to participate, and those who did not provide consent.

### 4.4. Knowledge, Attitudes, and Practices Survey Development

The questionnaires were designed to cover a range of topics, including demographic information, an assessment of AMU conditions, and an evaluation of KAP connected with AMU and AMR among farmers. Most questions in the questionnaire were multiple-choice. Demographic information included age, gender, educational level, training in AMR, training in AMU, and years of experience in chicken farming. AMU-related questions addressed various aspects of poultry management, such as the types of antimicrobials used, their sources, storage practices, and overall farm management, including flock size, bird health, the specific antimicrobial used by farmers, and the recurrence of illness. Knowledge-related questions centered on AMU, antibiotic withdrawal periods, AMR transmission, and government regulations concerning AMU. Regarding perceptions of AMU and AMR, the attitude section investigated factors such as the use of prescribed antimicrobials and instances of overdosing. Finally, the practice-related questions aimed to gather information on sensitivity testing, adherence to full courses of antimicrobials, and whether farmers consult veterinarians.

### 4.5. Sample Collection

#### Semi-Structured Interviews (SSI)

Data collection was done using a self-administered structured questionnaire facilitated by the Kobo Collect tool. The researcher administered the pretesting of the questionnaires, where 9 farmers were given the questionnaires to respond to and to see if the questions were relevant, clearly understood, and made sense before going for the data collection of the study. The questionnaires were checked and validated by experts to ensure the objectives of the study were answered. The reliability was checked by factor analysis of all knowledge, attitudes, and practices, and the results were above 0.7.

The farmers were interviewed voluntarily after being fully informed about the survey’s objectives, and verbal consent was obtained before participation. Each consenting poultry farmer received a structured questionnaire designed to assess their KAP regarding AMU and AMR. The questionnaire was developed in English and then translated into Tumbuka when collecting data, to ensure that all respondents were comfortable with the language used. Each interview lasted 20 to 30 min.

### 4.6. Data Management and Analysis

The questionnaire was stored online on KoboToolbox (https://www.kobotoolbox.org). The data were then exported into a Microsoft Excel worksheet 2016 for cleaning and management, and thereafter posted into STATA version 15 software, where all statistical analyses were done. Descriptive statistical analysis of the responses captured using questionnaires was performed using Microsoft Excel worksheet 2016. STATA version 15 was used to compute correlation coefficients of the independent variables, such as age, sex, marital status, education level, years in practice, formal training in AMU, and formal training in AMR, against their dependent variables, such as knowledge, attitude, and practices scores. After computing mean scores, linear regression was used to examine associations between knowledge, attitudes, and practice scores. The assessment of knowledge, attitude, and practice was performed with a scoring system. The KAP scores of the participants were calculated as the sum of correct responses to each question. The correct response was scored as 1 and the wrong response as 0. Data coding was done for the variables (knowledge, attitude and practice); each correct response was given a score of 1, while a wrong response was given a score of 0. Each respondent could obtain a score of 0–11 for knowledge, 0–13 for attitude, and 0–9 for practices.

The study used mean scores with their standard error (SE) as a cut-off point because there was no cut-off point to assess poor and better/good knowledge, attitudes, and practices. Scores above and equal to the mean were regarded as better knowledge, practice, and a positive attitude, while scores below the mean were considered as low knowledge, practice, and a positive attitude. After performing mean scores, linear regression was used to compare the knowledge, attitudes, and practice scores and demographic variables. The linear regression was validated by checking the Variance Inflation Factor (VIF). The VIF for all demographic variables vs. knowledge, attitudes and practices scores was between 1.02 and 1.3. All had no problem with multicollinearity; hence, the analysis was suitable for the test. Pearson’s correlation test was further used to compare whether there was a relationship among knowledge, attitudes, and practices. As per the criteria by [47], the correlations were classified as 0–0.25 = weak correlation, 0.25–0.5 = fair correlation, and above 0.5 = good correlation.

In all the analyses, alpha was set at *p* ≤ 0.05 for significance differences. Sensitivity analysis was used in data management to deal with confounding variables.

### 4.7. Ethical Approval

Ethical approval was obtained from the Department of Animal Health and Livestock (DAHLD) in Malawi (Ref: NO DAHLD/AHS/01/2025/02) dated 28 January. Informed oral consent was obtained from every chicken farmer before the interview. Before the interviews, the study objectives, interview process, and the usage of the data were fully understood by the participants.

## 5. Conclusions and Recommendations

This study found that broiler farmers had low levels of knowledge, attitudes and practices on AMU and AMR. Using demographic variables, it has been observed that farmers who received training on AMU and AMR demonstrated good knowledge and practices related to AMU and AMR.

Factors such as easy access to antibiotics, inadequate guidance from veterinary professionals on proper administration and antibiotic selection, limited health and national policies related to AMU and AMR, and poor government oversight of antimicrobial acquisition and use can contribute to the emergence of AMR in chicken production.

Some demographic variables, such as gender and education level, did not have an impact on AMU and AMR. This means that when the government implements policies, they need to include training among farmers on AMU and AMR. The results highlight the need for further research to understand the interactions between veterinarians and farmers, particularly regarding the use of antibiotics in chicken farming. Filling the gaps in these interactions will inform antimicrobial stewardship training which will prevent and control antimicrobial resistance in broiler chickens.

## Figures and Tables

**Table 1:** Demographic characteristics (n = 89).

Variable	Categories	Frequency (%)
Gender	Male	51 (57%)
	Female	38 (43%)
Age group	20–30	13 (14.6%)
	31–40	27 (30.3%)
	41–50	21 (23.6%)
	Above 50	28 (31.5%)
Marital status	Single	15 (17%)
	Married	74 (83%)
Education level	No formal education	2 (2%)
	Primary	11 (12%)
	Secondary	62 (70%)
	Tertiary	14 (16%)
Number of years in poultry practice	0–1	10 (11%)
	1–4	29 (32%)
	4–10	21 (24%)
	More than 10	29 (32%)
Formal training of AMR	No	86 (97%
	Yes	3 (3%)
Formal training of AMU	No	69 (78%)
	Yes	20 (22%)

**Table 2:** Prescription of antimicrobial information.

Variable	Category	Frequency (%)
Who has the authority to write a prescription	Registered veterinarian	68 (76%)
	Not sure	9 (10%)
	Vet shop owners	6 (7%)
	Anyone	6 (7%)
Are prescriptions written	No	80 (90%)
	Yes	9 (10%)
Do the Veterinary medicine stores request prescriptions for some antimicrobials like antibiotics?	No	87 (97%)
	Yes	2 (2%)

**Table 3:** Classes of antibiotics used by farmers in the study area.

Class of Antibiotics	Antibiotics Used by Farmers in Poultry Farming	n (%) Farmers
Tetracycline	Alamycine, Trimovate, oxyfarm, Trimo farm, egocin, oxysol, tetracycline, limoxin,	56 (63)
Quinolone	Arysel, interflox, livita, enflolaxin, limox.	15 (16.8)
Trimethoprim/sulphamethzole	Intertrim, Vitamed, Trisulmycine, Bactrim, co-trimozaxole	20 (22.5)
Penicillin	Contrivet (amoxicillin), democycline, piperacillin,	5 (5.6)
Aminoglycosides	Neomycin, biosole	3 (3.4)
Cephalosporin’s	Vetox	1 (1)
Tylosin	Dawa tylodoxy, Batylocin	2 (2.2)

**Table 4:** Knowledge of chicken farmers on AMU and AMR.

Variable	Response (N/%)	
	Yes	No
Do you know that antimicrobial resistance is a serious public health problem?	22 (25)	67 (75)
Does the Government of Malawi have a policy/framework for antimicrobial use in animals?	32 (36)	57 (64)
Heard of AMR	84 (94)	5 (6)
Heard of AMU	87 (98)	2 (2)
Do you know that antimicrobials can be passed on to humans through the consumption of chicken products?	85 (96)	4 (4)
Do you know that inappropriate use of antimicrobials can lead to the development of the AMR?	81 (91)	8 (9)
Do you know that it is wrong to sell your animal products (meat)before the withdrawal period is over after administering?	86 (97)	3 (3)
Do you know that antimicrobial residues in poultry animals could be hazardous for public health?	84 (94)	5 (6)
Do you know that the use of antimicrobials in feed formulation is inappropriate?	24 (27)	65 (73)
Do you know who controls the sale of antimicrobials?	56 (63)	33 (37)
Do you know that lack of control in the sales of antimicrobials contributes to AMR?	62 (70)	27 (30)

**Table 5:** Correlation of independent variables with knowledge scores.

Variable	*t*-Test	Significance
Age range group	−0.04	0.969
Gender	2.65	0.010
Marital status	0.42	0.678
Years in poultry farming	0.03	0.978
Education level	54.64	0.000
AMU training	2.59	0.011
AMR training	3.27	0.002

**Table 6:** Attitudes of farmers on AMU and AMR.

Variable	Strongly Agree	Agree	Neutral	Disagree	Strongly Disagree	
Antimicrobials are safe, so they are commonly used in human and animals.	87 (98%)	0 (0)	1 (1%)	1 (1%)	0 (0%)	
Antimicrobials are needed for the treatment of any type of illness in animals.	81 (91%)	2 (2%)	3 (3%)	2 (2%)	1 (1%)	
It is better to make sure that animals are cured by broad-spectrum antimicrobials?	83 (93%)		2 (2%)	3 (3%)	1 (1%)	0 (0%)
Antimicrobials are needed to prevent only serious illness?	73 (82%)		2 (2%)	10 (11%)	2 (2%)	2 (2%)
Non-prescribed antimicrobial sales should be prohibited?	84 (94%)		3 (3%)	0 (0%)	0 (0%)	2 (2%)
When weather/seasons change, antimicrobials are needed for animals?	51 (57%)		4 (4%)	26 (29%)	2 (2%)	6 (6%)
When animals have a fever/cold, antimicrobials are needed?	56 (62)		19 (21%)	6 (7%)	4 (4%)	4 (4%)
Once the animals are cured, it’s important to stop the dosage so that they are kept safe in case the animals get sick again in the future and can be re-used?	70 (79%)		6 (7%)	10 (11%)	3 (3%)	0 (0%)
It’s safe to eat an animal if it dies in the course of treatment	2 (2%)		0 (0%)	4 (4%)	14 (16%)	69 (78%)
Overdosing can lead to AMR	29 (33%)		2 (2%)	11 (12%)	12 (13%)	35 (39%)
Missing a dose of antimicrobials may contribute to antimicrobial resistance?	85 (96.5%)		1 (1%)	1 (1%)	1 (1%)	2 (2%)
Vaccination can reduce the use of antimicrobials in animal farms?	55 (62%)		2 (2%)	25 (28)	1 (1%)	6 (7%)
Antimicrobials should be added to feed at any time to prevent animals from becoming sick.	17 (19%)		3 (3%)	22 (25%)	16 (18%)	31 (35%)
Expired antimicrobial can be given to animals when they are sick rather than waste/dispose it?	0 (0%)	5 (6%)	0	14 (16%)	70 (79%)	

**Table 7:** Correlation of independent variables with attitudes scores.

Variable	*t*-Test	Significance
Age range group	1.14	0.257
Gender	−0.55	0.585
Marital status	−0.67	0.508
Years in practice	−0.95	0.345
Education level	−0.65	0.519
Training AMU	1.00	0.319
Training AMR	0.95	0.346

**Table 8:** Practices of chicken farmers on AMU and AMR.

Variable	Category	Frequency (%)
What do you do when animals sick	Treat without sensitivity tests	84 (94%)
	Treat after carrying out sensitivity tests at the laboratory	5 (6%)
Whom do you consult when animals are sick?	No body	46 (51%)
	Assistant veterinarian	25 (28%)
	Farmer technician	10 (11%)
	Veterinarian	8 (8.99)
Whom do you consult for selection of antimicrobials and its dosage?	No body	56 (63%)
	Assistant veterinarian	23 (26%)
	Farmer technician	6 (7%)
	Veterinarian	4 (4%)
Whom do you consult for the preparation and administration of antibiotics	No body	61 (69%)
	Assistant veterinary	17 (19%)
	Farmer technician	7 (8%)
	Veterinary	4 (4%)
Do you allow your animals to complete the entire course of antimicrobials as prescribed by the veterinarian?	No	8 (9)
	Yes	81 (91%)
Do you check the expiry date of the antimicrobials before administering them/selling them	No	14 (16%)
	Yes	75 (84%)
Do you continue issuing antibiotics when the symptoms disappear	No	82 (92%)
	Yes	7 (8%)
What do you do when you realize that the antimicrobials have expired?	Burn/bury them	82 (92%)
	Throw them	57 (64%)
	Put them in animal feed	5 (6%)
	Return to veterinary labs	2 (2.2%)
	Use them	2 (2.2%)
Do you increase the dose and frequency of antimicrobials when there are clinical symptoms?	No	17 (19%)
	Yes	72 (80%)

**Table 9:** Correlation of independent variables with practices scores.

Variable	*t*-Test	Significance
Age range group	−0.44	0.659
Gender	0.87	0.388
Marital status	−0.54	0.591
Years in practice	−0.53	0.596
Education level	0.24	0.809
AMU training	1.32	0.191
AMR training	2.29	0.024

**Table 10:** Correlation of KAP of farmers on AMU and AMR.

Variables	Coefficient	*p*-Value
Knowledge and attitudes	0.2097	0.0486
Knowledge and practices	0.0732	0.4957
Attitudes and practices	−0.0539	0.6162

## Data Availability

The majority of the data generated in this study are presented in the results section through tables and figures. Additional data can be obtained from the corresponding authors upon reasonable request.
